# A Targetron-Recombinase System for Large-Scale Genome Engineering of Clostridia

**DOI:** 10.1128/mSphere.00710-19

**Published:** 2019-12-11

**Authors:** Tristan Cerisy, William Rostain, Audam Chhun, Magali Boutard, Marcel Salanoubat, Andrew C. Tolonen

**Affiliations:** a Génomique Métabolique, Genoscope, Institut François Jacob, CEA, CNRS, Univ Evry, Université Paris-Saclay, Évry, France; University of Wisconsin-Madison

**Keywords:** clostridia, engineering, prophage, recombinase

## Abstract

Clostridia are a group of Gram-positive anaerobic bacteria of medical and industrial importance for which limited genetic methods are available. Here, we demonstrate an approach to make large genomic deletions and insertions in the model Clostridium phytofermentans by combining designed group II introns (targetrons) and Cre recombinase. We apply these methods to delete a 50-gene prophage island by programming targetrons to position markerless *lox66* and *lox71* sites, which mediate deletion of the intervening 39-kb DNA region using Cre recombinase. Gene expression and growth of the deletion strain showed that the prophage genes contribute to fitness on nonpreferred carbon sources. We also inserted an inducible fluorescent reporter gene into a neutral genomic site by recombination-mediated cassette exchange (RMCE) between genomic and plasmid-based tandem *lox* sites bearing heterospecific spacers to prevent intracassette recombination. These approaches generally enable facile markerless genome engineering in clostridia to study their genome structure and regulation.

**IMPORTANCE** Clostridia are anaerobic bacteria with important roles in intestinal and soil microbiomes. The inability to experimentally modify the genomes of clostridia has limited their study and application in biotechnology. Here, we developed a targetron-recombinase system to efficiently make large targeted genomic deletions and insertions using the model Clostridium phytofermentans. We applied this approach to reveal the importance of a prophage to host fitness and introduce an inducible reporter by recombination-mediated cassette exchange.

## Abstract (precis)

Clostridia are anaerobic bacteria with important roles in intestinal and soil microbiomes. The inability to experimentally modify the genomes of clostridia has limited their study and application in biotechnology. Here, we developed a targetron-recombinase system to efficiently make large targeted genomic deletions and insertions using the model Clostridium phytofermentans. We applied this approach to reveal the importance of a prophage to host fitness and introduce an inducible reporter by recombination-mediated cassette exchange.

## INTRODUCTION

The clostridia are Gram-positive obligately anaerobic bacteria that include human pathogens as well as plant-fermenting species critical for healthy functioning of soil and gut microbiomes. *Clostridium* (*Lachnoclostridium*) *phytofermentans* ISDg (1) is a model plant-fermenting *Clostridium* that breaks down plant biomass using numerous carbohydrate-active enzymes (CAZymes) and ferments the resulting hexose and pentose sugars into ethanol, hydrogen, and acetate (2–4). *C. phytofermentans* is a member of the *Lachnospiraceae* family that is abundant in soil (5), dominates the rumen (6), and includes human gut commensals that play important roles in nutrition and intestinal health (7). Because of their ability to directly ferment lignocellulose, plant-fermenting clostridia have industrial potential for the transformation of plant biomass into value-added chemicals. Clostridia have long been the focus of study due to their diverse importance to human health, industry, and the environment (8). However, a lack of methods for genetic manipulation of clostridia has hindered their application in biotechnology and our ability to study the structure and function of their genomes.

The development of targetrons based on the Lactococcus lactis Ll.LtrB group II intron (9) enabled targeted chromosome insertions in *C. phytofermentans* (10) and other clostridia (11). Targetrons are designed group II introns that can be customized to insert into specific DNA sequences by a retrohoming mechanism with efficiencies approaching 100% of cells (12), obviating the need for antibiotic resistance markers to select for their insertion. Domain IV of the intron can be modified to carry cargo DNA such as single or two tandem *lox* sites (13). *Lox* sites are 34-bp elements of two 13-bp palindromic arms separated by an 8-bp spacer that are recognized by Cre, a recombinase that does not require host-encoded factors (14). Cre has previously been applied in clostridia to excise an antibiotic resistance gene integrated by homologous recombination (15). Modified *lox* sequences expand the utility of the Cre/*lox* system. For example, *lox66* and *lox71* sites each contain arm mutations such that their recombination results in a *lox72* site with two mutant arms that is no longer recognized by Cre (16). Orthogonal spacer mutants such as *lox511* and *loxFAS* do not recombine with each other (17), permitting simultaneous noninterfering *lox* recombinations for recombination-mediated cassette exchange (RMCE). RMCE is a method by which a plasmid and genomic cassette can be exchanged by recombination mediated by a site-specific recombinase such as Cre (18).

**FIG 1:**
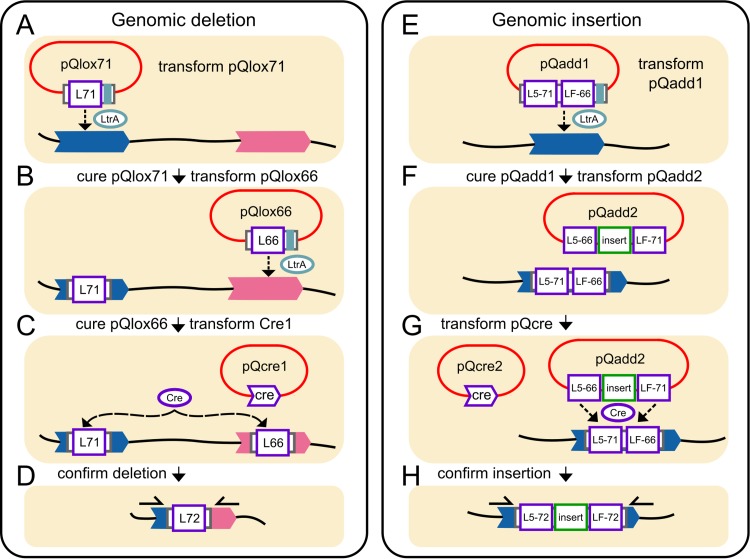
Overview of genomic deletion and insertion in *C. phytofermentans*. (A) pQlox71 is introduced for genomic insertion of a *lox71* (L71) site using the LtrA protein encoded by the targetron. (B) pQlox71 is cured and pQlox66 is introduced for genomic insertion of a *lox66* (L66) site. (C) pQlox66 is cured, and pQcre1 is introduced for Cre-mediated recombination to delete the sequence between the *lox66* and *lox71* sites. (D) In the resulting strain, the deletion and *lox72* site are confirmed by PCR (arrows show primers). (E) pQadd1 is introduced for genomic delivery of a *lox511*/*71* (L5-71) and *loxFAS*/*66* (LF-66) cassette into the genome. (F) pQadd1 is cured and pQadd2 is introduced, bearing the desired insertion sequence flanked by *lox511*/*66* (L5-66) and *loxFAS*/*71* (LF-71) sites. (G) pQcre2 is introduced for Cre-mediated RMCE. (H) The resulting strain has a genomic copy of the insert sequence flanked by *lox511*/*72* (L5-72) and *loxFAS*/*72* (LF-72) sites in the genome, which is confirmed by PCR (arrows show primers).

Here, we describe a way to make large precise deletions (Fig. 1A to D) and insertions (Fig. 1E to H) in clostridial genomes using *C. phytofermentans* as a model. Targetrons and Cre recombinase have been used together to make genomic insertions in Escherichia coli and deletions in E. coli and Staphylococcus aureus (13), showing that these tools can be used together in Gram-negative and Gram-positive bacteria. We developed a targetron-recombinase system for clostridia and applied it to excise 50 genes (*cphy2944* to *cphy2993*) comprising a 39-kb prophage region in the *C. phytofermentans* genome, which we chose because mobile elements such as prophages often reduce genomic stability and their removal increases fitness in other Gram-positive bacteria (19). We also inserted an anaerobic flavin mononucleotide-based fluorescent protein (20) into the *C. phytofermentans* genome by RMCE between genomic and plasmid-based tandem *lox* cassettes and demonstrated that it acts as a carbon source-inducible reporter. We discuss how these methods are an important component of an emerging suite of tools to engineer clostridial genomes.

## RESULTS

### Plasmids for genomic deletion and insertion.

Our strategy to make targeted deletions (Fig. 1A to D) and insertions (Fig. 1E to H) in the *C. phytofermentans* genome is based on sequential introduction and removal of conditionally replicating plasmids carrying *lox* sites or Cre recombinase. We constructed plasmids for targetron-mediated delivery of *lox* sites by inserting the *lox* cassettes into the unique MluI site in domain IV of the intron, a site that supports DNA insertions while retaining targetron activity (13). Targetrons can be programmed to insert into the genome in either orientation, but both *lox* linkers must be in the same relative orientation in the genome for Cre to delete the intervening region (Fig. 1C). Similarly, RMCE requires two Cre-based recombinations between tandem *lox* sites whose orientation determines that of the inserted DNA (Fig. 1E to H). We thus constructed *lox-*containing targetrons in pQint (10) with either orientation of *lox* sites: *lox71* (pQlox71F and pQlox71R) and *lox66* (pQlox66F and pQlox66R) for genome deletions and a tandem *lox511*/*71*-*loxFAS*/*66* cassette (pQadd1F and pQadd1R) for genome insertion by RMCE (Table 1; see also Fig. S1 in the supplemental material). These plasmids all contain the erythromycin resistance gene from Streptococcus pneumoniae Tn*1545* (21) for selection in E. coli and *C. phytofermentans* and the pAMβ1 origin that replicates stably in *C. phytofermentans* but can be cured by serial transfer in liquid medium lacking antibiotics (10).

**TABLE 1:** Plasmids and strains used in this study

Plasmid or Strain	Description	Source or reference
Plasmids		
pAT19	*erm* (erythromycin resistance), pAMβ1 origin, pUC origin, oriT	21
pQint	pAT19 with targetron (Ll.LtrB-deltaORF intron, *ltrA*) expressed from P3558 promoter	10
pRAB1	Source of P*pagA*-*cre* cassette	22
pRK24	RP4 conjugal genes, Tet^r^, Amp^r^	10
pMTL82351	*aad9* (spectinomycin resistance), pBP1 origin, colEI origin, oriT	CHAIN Biotech
pMTC6	MlsR, Amp^r^, E. coli*-Clostridium* shuttle vector. P*pFbFPm*, *lac* operator, *thl* promoter, *thl* terminator	32
pQlox71F, pQlox71R	pQint with *lox71* in either the forward (F) or reverse (R) orientation inserted into MluI site	This study, Addgene 135655, 135656
pQlox66F, pQlox66R	pQint with *lox66* in either the forward (F) or reverse (R) orientation inserted into MluI site	This study, Addgene 135657, 135658
pQcre1	pAT19 with P*pagA*-*cre* inserted between EcoRI and XbaI sites	This study, Addgene 135659
pQcre2	pMTL82351 with P*pagA*-*cre* cassette from pQcre1 inserted in EcoRI site	This study
pQadd1F, pQadd1R	pQint with *lox511*/*71* and *loxFAS*/*66* cassette inserted into MluI site	This study, Addgene 135660, 135661
pQadd2	pAT19 with *lox511*/*66-loxFAS*/*71* cassette inserted between the PstI and EcoRI sites	This study. Addgene 135662
pQadd2_P3368_FbFP	pQadd2 with P3668-*FbFP* inserted between SpeI and XhoI sites of the *lox* cassette	This study
Strains		
E. coli 1100-2	*mcrA0*, *endA1*, mcrB9999 strain used as a conjugal donor	10
E. coli NEB 5-alpha	Competent E. coli cells used for gene cloning	New England BioLabs
*C. phytofermentans* ISDg	Reference strain	ATCC 700394
*C. phytofermentans* IntR2944	*cphy2944*::*lox71*F.int2944.591s	This study
*C. phytofermentans* IntF2944	*cphy2944*::*lox71*R.int2944.526a	This study
*C. phytofermentans* DI-AS	*cphy2944*::*lox71*R.int2944.591s, *cphy2993*::*lox66*F.int2993.177a	This study
*C. phytofermentans* DI-SS	*cphy2944*::*lox71*F.int2944.526a*, cphy2993*::*lox66*F.int2993.177a	This study
*C. phytofermentans* Del-AS	DI-AS-derived *cphy2944-cphy2993* deletion strain with palindromic scar	This study
*C. phytofermentans* Del-SS	DI-SS-derived *cphy2944-cphy2993* deletion strain with *lox72* site	This study
*C. phytofermentans* Int1575	*cphy1575*::*lox511*/*71*_*loxFAS*/*66*.96a	This study
*C. phytofermentans* Int1575-FbFP	*cphy1575*::*lox511*/*72*_P3368-*FbFP*_*loxFAS*/*72*.96a	This study

10.1128/mSphere.00710-19.1 FIG S1 Plasmids for genomic deletion and genomic insertion by intron-mediated delivery of *lox* cassettes and expression of Cre recombinase. (A) The introns of pQlox71F and pQlox66F carry *lox71* or *lox66* sites inserted in the forward orientation; pQlox71R and pQlox66R (not shown) carry *lox* sites in the reverse orientation. pQcre1 expresses cre using the P*pagA* promoter from B. anthracis. (B) Plasmids for genomic insertion. The intron of pQadd1R carries a *lox511*/*71*-*loxFAS*/*66* cassette in the reverse orientation; pQadd1F (not shown) carries the same lox sites in the other orientation within the intron. pQadd2 bears a *lox511*/*66*-*loxFAS*/*71* cassette with central SpeI and XhoI sites into which cargo DNA for genomic insertion can be cloned. All plasmids are built using the pQint backbone except for pQcre2, which has a pBP1 origin and spectinomycin resistance gene to facilitate cotransformation with pQadd2. Download FIG S1, EPS file, 0.2 MB. Copyright © 2019 Cerisy et al. 2019 Cerisy et al. This content is distributed under the terms of the Creative Commons Attribution 4.0 International license.

To express Cre recombinase in *C. phytofermentans* for genomic deletion between *lox71* and *lox66* sites, we constructed pQcre1 (Fig. S1; Table 1) in which *cre* is expressed from the Bacillus anthracis P*pagA* promoter, a well-characterized constitutive promoter that is widely functional in Gram-positive bacteria (22). Initially, we attempted to deliver a tandem *lox* cassette and *cre* on a single plasmid for RMCE but were unable to construct such a plasmid likely because of interactions between Cre and the *lox* cassette, requiring us to cotransform *C. phytofermentans* with separate plasmids bearing the *lox* cassette and *cre* gene. We found that the pBP1 origin from Clostridium botulinum (23) replicates stably in *C. phytofermentans* and can be cured using the same methods as for pAMβ1 plasmids and that the *aad9* adenyltransferase gene from Enterococcus faecalis (24) confers spectinomycin resistance in *C. phytofermentans*. Moreover, we found that plasmids bearing pAMβ1 and pBP1 origins can be simultaneously maintained in the same *C. phytofermentans* cell, provided they have different antibiotic resistance markers. Thus, we constructed pQcre2 in which the P*pagA*-*cre* cassette from pQcre1 was inserted into pMTL82351 (Table 1), which carries the pBP1 origin and *aad9*.

### Targetrons position genomic *lox* sites.

**FIG 2:**
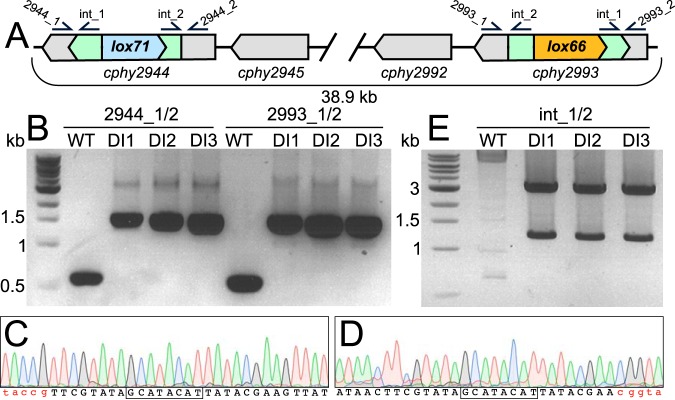
Construction of a *C. phytofermentans* strain with targetron-mediated insertion of a *lox71* site in *cphy2944* and a *lox66* site in *cphy2993*. (A) Genome region with the *lox*-containing targetron insertions in *cphy2944* and *cphy2993*. Positions of primers used in panels B and E are shown. (B) PCR confirmation of *lox* insertions into *cphy2944* (primers 2944_1/2) and *cphy2993* (primers 2993_1/2) in 3 DI-AS isolates (DI1 to DI3). DNA chromatograms from DI1 of the *lox71* site in *cphy2944* (C) and the *lox66* site in *cphy2993* (D) with the 8-bp central spacer outlined and arm mutations relative to *loxP* shown in red. (E) Inverse PCR (primers int_1/2) shows the 3 DI-AS isolates (DI1 to DI3) contain only the 2 expected genomic targetron insertions. The 3.5-kb band corresponds to the targetron insertion in *cphy2944* and the 1.3-kb band to the insertion in *cphy2993*.

Both *lox* sites must be in the same orientation for Cre-based recombination to result in deletion of the intervening DNA (25). To excise the 39-kb prophage region (*cphy2944-2993*) in the *C. phytofermentans* genome (Fig. 2A), pQlox71R was modified to insert a *lox71* targetron at 591 bp from the *cphy2944* start codon in the sense direction relative to gene transcription, yielding pQlox71R.2944. Plasmid pQlox66F.2993 was similarly built to insert a *lox66* targetron in the antisense orientation at 177 bp from the start of *cphy2993*. Both *cphy2944* and *cphy2993* are transcribed on the reverse strand of the genome, such that the *lox71* and *lox66* sites inserted by pQlox71R.2944 and pQlox66F.2993 are in the same direction the genome (Fig. 2A).

Conjugal transfer of *lox71* and *lox66* targetron plasmids into *C. phytofermentans* consistently yielded 10 to 20 transconjugant colonies, of which 80% to 100% contained the expected targetron insertion, indicating that the efficiency of delivery and integration of *lox*-containing targetrons is similar to that in previous targetrons studies in *C. phytofermentans* (10, 26, 27). Following sequential delivery and curing of pQlox71R.2944 and pQlox66F.2993, PCR amplification of *cphy2944* and *cphy2993* showed targetron insertions in both genes (Fig. 2B), and sequencing of these PCR products established the presence of the expected genomic *lox71* in *cphy2944* (Fig. 2C) and *lox66* in *cphy2993* (Fig. 2D). We refer to this double insertion strain as DI-AS (Table 1). Off-site targetron insertions are common (27), which would result in unexpected recombinations. Thus, we applied an inverse PCR assay (27) using primers that anneal to the targetron in order to quantify the number and sequence of genomic targetron insertions. This assay confirmed that DI-AS contains both expected genomic targetrons without any additional off-site insertions (Fig. 2E).

### Cre-mediated prophage deletion.

**FIG 3:**
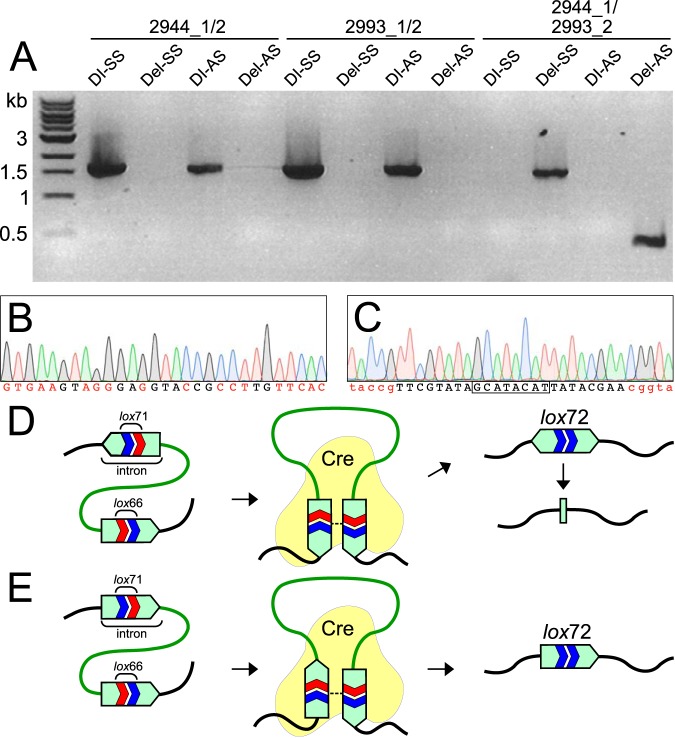
Cre-mediated genomic deletion in *C. phytofermentans*. (A) PCR of the *cphy2944* gene (primers 2944_1/2), the *cphy2993* gene (primers 2993_1/2), and the 38.9-kb genomic region (primers 2944_1/2993_2) before pQcre1 transformation (strains DI-SS and DI-AS) and after pQcre1 transformation (strains Del-SS and Del-AS). (B) DNA chromatogram of the intron fragment in strain Del-AS with palindromic positions shown in red. (C) DNA chromatogram of the *lox72* site in Del-SS with 8-bp central spacer outlined and arm mutations relative to *lox*P in red. Model of how Cre-mediated deletion of the genomic region between *lox71* and *lox66* sites results in a bidirectional intron that recombines into a 28-bp intron fragment lacking a *lox72* site in strain Del-AS (D) and a unidirectional intron containing a *lox72* site in strain Del-SS (E).

We introduced Cre recombinase into *C. phytofermentans* strain DI-AS by conjugal transfer of pQcre1 and diagnosed the Cre-mediated deletion using 3 PCR amplifications: amplicons spanning each genomic *lox* site and an amplicon spanning the genomic region between the two *lox* sites. Cre-mediated recombination was highly efficient in *C. phytofermentans* such that when DI-AS was transformed with pQcre1, all colonies yielded a product using primers 2944_1/2993_2 spanning the region between *lox71* and *lox66* and no colonies yielded PCR products using primers flanking each of the individual *lox* sites (2944_1/2 and 2993_1/2) (Fig. 3A). We refer to the strain with a Cre-mediated deletion as Del-AS (Table 1). While all 10 Del-AS transconjugants yielded PCR products with primers 2944_1/2993_2, the product was 400 bp rather than the expected 1.5-kb product (Fig. 3A). The sequence of this PCR product revealed that all that remained of the expected *lox72*-containing targetron was a palindromic fragment corresponding to the first 28 bp of the targetron (Fig. 3B).

Cre-mediated recombination between *lox* sites in opposing targetrons produces a targetron fragment consisting of a 500-bp inverted repeat that may be unstable. We tested if the relative orientation of the genomic targetrons influences the final product of Cre recombination by generating a second *C. phytofermentans* strain with both targetrons in the same orientation. To this end, we modified the *lox71*-targetron of pQlox71F.2944 to insert in *cphy2944* in the sense orientation with respect to transcription at 526 bp from the gene start. As the orientation of both the intron targeting and *lox71* are reversed relative to that in pQlox71R.2944, the orientation of *lox71* in the genome is preserved. We constructed a second double insertion strain, DI-SS (Table 1), with both genomic targetrons in the same orientation by sequential delivery and curing pQlox71F.2944 and pQlox66F.2993 using the same procedures as for DI-AS. Following delivery of pQcre1 to DI-SS, we observed by PCR using primers 2944_1/2993_2 that all 10 transconjugants yielded a 1.5-kb product (Fig. 3A) whose sequence confirmed the expected recombination resulting in a single targetron containing a *lox72* site (Fig. 3C). We refer to this strain as Del-SS (Table 1). We thus conclude the Cre-mediated deletion in Del-AS results in an inverted repeat that is excised by homologous recombination to leave only a short palindromic targetron fragment (Fig. 3D), whereas the intact intron containing a *lox72* site in Del-SS is stable in the genome (Fig. 3E).

### Prophage excision reduces growth on nonpreferred carbon sources.

**FIG 4:**
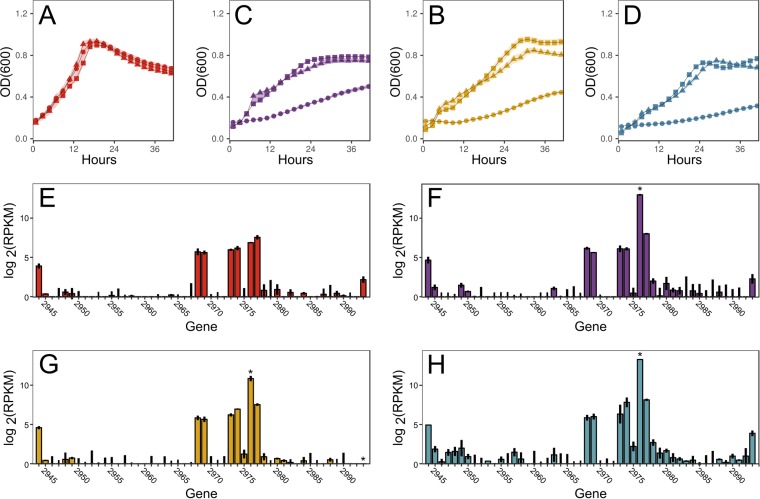
Growth of *C. phytofermentans* WT (■), DI-SS (▲), and Del-SS (●) strains on glucose (A), cellobiose (B), xylose (C), and galactan (D). Data points are means from 4 cultures; shaded areas show ± standard deviations (SDs). mRNA expression measured by RNA-seq of *cphy2944-cphy2993* in *C. phytofermentans* WT on glucose (E), cellobiose (F), xylose (G), and galactan (H). Bars show mean log_2_(RPKM) ± SD from duplicate cultures; stars show genes differentially expressed on other carbon sources relative to glucose. RNA-seq measurements and differential expression statistics are based on a previous study (3).

Wild-type and DI-SS strains grow similarly on monosaccharides (glucose and xylose), a disaccharide (cellobiose), and a polysaccharide (galactan) (Fig. 4A to D), indicating that targetron insertions in *cphy2944* (phage cell wall peptidase) and *cphy2993* (phage DNA-binding protein) do not affect growth under these conditions. In contrast, growth of Del-SS was significantly reduced relative to that of the wild-type (WT) on carbon sources other than glucose (Fig. 4A to D), suggesting that the prophage contributes to cell fitness. The organization and sequences of the 50 genes in the *C. phytofermentans* prophage region are similar to those in Bacillus subtilis bacteriophage SPP1, which has a linear, double-stranded 44-kb DNA genome with all genes transcribed in the same direction (28). While it is unknown if this *C. phytofermentans* prophage can form lytic particles, it harbors genes for the major phage functions: bacterial cell wall binding and degradation, phage tail assembly, phage head assembly, and DNA synthesis/replication (see Fig. S2). However, the contribution of these genes to cell fitness on nonglucose carbon sources is not evident from gene annotations.

10.1128/mSphere.00710-19.2 FIG S2 Region of the *C. phytofermentans* genome harboring a 39-kb prophage comprised of the 50 genes *cphy2944-cphy2993*. Gene orientations and annotations are shown with colors describing phage genes (red), conserved hypotheticals (blue/black), and the two divergently transcribed genes *cphy2975-cphy2976* (purple). The positions of the *lox71* insertion in *cphy2944* and the *lox66* insertion in *cphy2993* are shown (yellow). Download FIG S2, EPS file, 0.1 MB. Copyright © 2019 Cerisy et al. 2019 Cerisy et al. This content is distributed under the terms of the Creative Commons Attribution 4.0 International license.

We compared the mRNA expression in *C. phytofermentans* WT of the 50 prophage genes on these four carbon sources as measured by transcriptome sequencing (RNA-seq) (3), revealing that *cphy2976* transcription is upregulated 68-fold on cellobiose, 15-fold on xylose, and 86-fold on galactan relative to that on glucose (Fig. 4E to H). As such, *cphy2976* is among the top 10 most highly expressed genes in the cell on cellobiose and galactan. The *cphy2976* gene is cotranscribed with *cphy2975* (29) in an operon that opposes all other genes in the prophage region (Fig. S2). Neither gene encodes domains of known function nor has significant similarity to genes in the NCBI database, and so we cannot postulate their roles in the cell. However, our data indicate that deletion of these 50 putative prophage genes unexpectedly decreased *C. phytofermentans* growth on nonpreferred carbon sources, and the extremely high upregulation of *cphy2976* suggests that it plays an important role in cell fitness under these conditions.

### Genomic insertion by RMCE.

**FIG 5:**
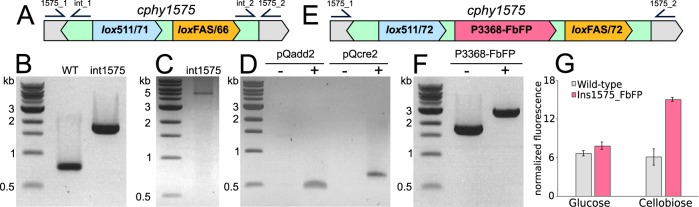
Genomic insertion in *C. phytofermentans* by RMCE. (A) Diagram of the *cphy1575* region in strain int1575 including positions of primers to confirm the targetron insertion in *cphy1575* (1575_1/2) and the number of genomic targetron insertions (int_1/2). (B) PCR of *cphy1575* in WT and int1575 (primers 1575_1/2) strains shows insertion of the targetron containing the *lox511*/71_*loxFAS*/*66* cassette in int1575. (C) Inverse PCR of int1575 genomic DNA (primers int_1/2) shows int1575 contains a single targetron insertion. The 5.1-kb band corresponds to the expected targetron insertion in *cphy1575*. (D) Plasmids pQadd2 and pQcre2 can be simultaneously maintained in strain int1575. PCR compares int1575 before plasmid transfer (− for pQAdd1 and pQcre2) and after transfer of both plasmids (+ for pQadd1 and pQcre2) using primers to amplify the plasmid origins of replication. (E) Diagram of *cphy1575* in int1575_FbFP after RMCE to insert the P3368-*FbFP* cassette. (F) PCR of *cphy1575* in int1575 (−) and int1575_FbFP (+) shows insertion of the P3368-*FbFP* cassette in int1575_FbFP. (G) Fluorescence (448/20-nm excitation, 495/20-nm emission) of wild-type and int1575_FbFP strains shows cellobiose-inducible FbFP expression. Bars show mean fluorescence normalized to cell density (OD_600_) from triplicate cultures ± SDs.

To effectuate targeted genomic insertions in *C. phytofermentans* by RMCE, the targetron of pQadd1R (Fig. S1) was customized to insert tandem *lox* sites with incompatible linkers (*lox511*/*71* and *loxFAS*/*66*) at 96 bp from the start of *cphy1575* (Fig. 5A) to produce pQadd1R.1575. The *cphy1575* gene is homologous to B. subtilis AprX, a nonessential S8 subtilisin serine protease that degrades heterologous protein in stationary phase (30, 31). The *cphy1575* gene is contained in a cluster of 6 genes with 50% or greater amino acid similarity (*cphy1571* to *cphy1576*) that likely have overlapping function, further suggesting that inserting heterologous DNA into *cphy1575* would have minimal impact on the cell. We transformed *C. phytofermentans* with pQadd1R.1575, confirmed the insertion of the intron into *cphy1575* by PCR (Fig. 5B), and cured the plasmid to yield strain int1575 (Table 1). We confirmed that int1575 did not contain any additional off-site targetron insertions by inverse PCR, which yielded only the expected 5.1-kb band corresponding to the insertion in *cphy1575* (Fig. 5C).

pQadd2 contains tandem *lox511*/*66* and *loxFAS*/*71* sites (Fig. S1) to facilitate RMCE with the complementary cassette integrated in the genome of strain int1575 (Fig. 5A). The *lox* sites in pQadd2 are separated by SpeI and XhoI sites into which we cloned a version of the FbFP oxygen-independent green fluorescent protein from Pseudomonas putida that has been codon optimized for clostridia (32). The *FbFP* gene is expressed from the promoter of *cphy3368* (P3368), encoding a GH48 cellobiohydrolase (33) whose transcription is highly upregulated on cellobiose relative to that on glucose (3). The resulting pQadd2 plasmid bearing the P3368-*FbFP* cassette was called pQadd2.P3368-FbFP. We sequentially transformed strain int1575 with pQadd2.P3368-FbFP and pQcre2. As these plasmids have different origins of replication and antibiotic resistance, they can be maintained simultaneously in the *C. phytofermentans* cell (Fig. 5D).

Following transformation of strain 1575 with pQadd2.P3368-FbFP and pQcre2, PCR amplification of the *cphy1575* genomic region (Fig. 5E) of 10 transconjugants showed that they all contained the intended insertion of the P3368-*FbFP* cassette (Fig. 5F). The sequence of this PCR product confirmed the insertion of the P3368-*FbFP* cassette flanked by *lox511*/*72* and *loxFAS*/*72* sites. As such, Cre-based recombination for RMCE is highly efficient in *C. phytofermentans*, similarly to genome deletion using Cre. We cured the plasmids, yielding strain int1575_FbFP (Table 1), and compared its fluorescence to that of the WT. The fluorescence of WT and int1575_FbFP cells was similar when grown on glucose, whereas int1575_FbFP showed elevated fluorescence relative to that of the WT on cellobiose (Fig. 5G). These results show that FbFP functions as a fluorescent reporter in *C. phytofermentans* and that P3368 can be used to regulate transcription of heterologous genes in a cellobiose-inducible manner.

## DISCUSSION

This study describes a strategy to make genomic deletions and insertions by leveraging two well-characterized genetic tools that are efficient and independent of host factors. Targetrons delivered both single and tandem *lox* sites into the *C. phytofermentans* genome in more than 80% of transconjugants, showing that splicing *lox* sites into the targetron does not significantly reduce its efficiency. Further, Cre effectuated recombination between *lox* sites for genomic deletion and RMCE-mediated insertion in 100% of colonies. Targetrons and Cre recombinase have already been used separately in other clostridial species, supporting that this approach can be generally applied in clostridia. Unexpectedly, we found that if the two genomic targetrons are in opposite orientations, the *lox72* site and most of the targetron sequence are excised to leave only a short scar following Cre-mediated recombination. While this secondary recombination can be prevented by controlling the orientation of the *lox* sites or potentially by introducing two nonhomologous targetrons based on Ll.LtrB and EclV, it is potentially preferable to reduce the size of the scar relative to a *lox*-containing targetron.

Engineering of clostridia with streamlined genomes will enhance our ability to optimize the production of target molecules and understand the contributions of nonessential genes to cell fitness. Deletion of one or a few genes by double-crossover homologous recombination-based integration of an antibiotic resistance marker has been achieved in certain clostridial species (34–36). Moreover, CRISPR is proving to be an efficient way to select for clostridia with markerless, homologous recombination-mediated gene deletions by killing nonrecombinant cells using Cas nucleases (37–39). CRISPR methods can select for small genomic changes because Cas nucleases can be programmed to cleave any genomic site containing a PAM sequence (40). However, CRISPR methods are still limited by the probability of initially introducing the desired mutation by homologous recombination, because CRISPR-mediated DNA cleavage only modestly induces recombination in bacteria (40) and most bacterial taxa lack the proteins for nonhomologous end joining (41). As the efficiency of homologous recombination decreases exponentially with distance between recombination sites (42), CRISPR methods are generally confined to making genomic changes of a few kilobases or less. Furthermore, the double-stranded DNA cleavage activities of Cas nucleases are toxic, making them difficult to introduce into clostridial species with low transformation efficiencies (41). In contrast, targetrons and Cre-*lox* are independent of homologous recombination, do not cause double-stranded DNA breaks, and are efficient such that large changes can be easily made without selection.

Insertion of heterologous genes and pathways into clostridia is fundamental to being able to endow them with novel phenotypic and metabolic properties. The *C. phytofermentans* int1575 strain containing a genomic *lox511*/*71*-*loxFAS*/*66* cassette is a chassis that can be generally applied for facile RMCE-mediated integration of genes and pathways of interest. Genomic insertion by RMCE does not require selectable markers, and the length of the inserted DNA is only limited by that which can be cloned into pQadd2. A previous method for genomic insertion in clostridia termed allele-coupled exchange (ACE) links expression of a selectable marker gene to the formation of a double-crossover recombinant chromosome (43). ACE is advantageous relative to RMCE in permitting serial accumulation of larger insertion fragments but disadvantageous in requiring a uracil auxotrophy or genomic insertion of antibiotic resistance genes. In conclusion, the targetron-recombinase method described here expands our capabilities to engineer clostridia by enabling large markerless genomic deletions and insertions. This approach complements other recent technologies such as CRISPR and ACE to make a suite of tools to understand the biology of clostridia and exploit their usefulness in industry and medicine.

## MATERIALS AND METHODS

### Cell cultivation and conjugation.

*C. phytofermentans* ISDg (ATCC 700394) was cultured anaerobically at 30°C in GS2 medium (44) containing 3 g liter^−1^ of either glucose (G5767; Sigma), xylose (X3877; Sigma), cellobiose (C7252; Sigma), or galactan (P-GALLU; Megazyme). Growth on different carbon sources was measured in 400-μl cultures in 100-well microtiter plates (9502550; Bioscreen) that were sealed in the anaerobic chamber (2% H_2_, 98% N_2_) as previously described (45). Cell densities were measured using a Thermo Scientific Bioscreen C as the optical density at 600 nm (OD_600_) with 30 s of shaking before each reading.

Plasmids were introduced into *C. phytofermentans* by conjugal transfer from E. coli strain 1100-2 (pRK24) (10), and plasmids were maintained using erythromycin (200 μg ml^−1^ in liquid medium, 40 μg ml^−1^ in solid medium) or spectinomycin (600 μg ml^−1^ in liquid medium and solid medium). Following conjugation, ten transconjugant colonies were picked, and the presence of the plasmid was confirmed by PCR using primers pAMB1_1/2 for pAMβ1 origin plasmids and primers pBP1_1/2 for pBP1 origin plasmids. Plasmids were cured by five successive transfers at 1:100 dilution in 5 ml liquid medium lacking antibiotics. Plasmid loss was confirmed based on antibiotic sensitivity and lack of a PCR product. The expected intron insertions in the *C. phytofermentans* genome were confirmed by PCR and sequencing using primers flanking the genomic insertion site (primers are listed in Table S1 in the supplemental material).

10.1128/mSphere.00710-19.3 TABLE S1 Primers used in this study. Download Table S1, XLSX file, 0.01 MB. Copyright © 2019 Cerisy et al. 2019 Cerisy et al. This content is distributed under the terms of the Creative Commons Attribution 4.0 International license.

### Plasmid construction.

Enzymes were purchased from New England BioLabs, and cloning was performed using NEB 5-alpha Competent E. coli cells (C2987I; NEB). The *lox66* and *lox71* sites were cloned by annealing complementary 5′ phosphorylated oligonucleotides (L71_1/2 and L66_1/2) and cloning them into the unique MluI site in domain IV of the Ll.LtrB-ΔORF intron of pQint (10). To enable positioning of *lox* sites in either orientation in the genome relative to the intron insertion, *lox66* and *lox71* sites were cloned into pQint in both orientations yielding 4 plasmids: pQlox66F, pQlox66R, pQlox71F, and pQlox71R. Using crossover PCR, the pQlox71F intron was targeted to insert in the antisense orientation at 526 bp from the *cphy2944* start codon (pQlox71F.2944), the pQlox71R intron was targeted to insert at 591 bp from the *cphy2944* start codon (pQlox71R.2944), and the pQintL66F intron was targeted to insert in the antisense orientation at 177 bp from the start of *cphy2993* (pQlox66F.2993). pQcre1 was constructed by PCR amplifying the P*pagA*-*cre* cassette from pRAB1 (22) using primers PpagA-Cre_1/2 and inserting it between the EcoRI and XbaI sites of pAT19 (21). PCR and sequencing using primers OK_PpagA-Cre_1/2 confirmed the presence of the P*pagA*-*cre* cassette in pQcre1.

pQadd1F/R were constructed by annealing complementary 5′ phosphorylated oligonucleotides 2ML4_1/2 encoding a double *lox* cassette (*lox511*/*71* and *loxFAS*/*66*) and cloning it into the unique MluI site of pQint. The pQadd1R intron was subsequently targeted to insert into *cphy1575* in the antisense orientation at 96 bp from the start codon to make plasmid pQadd1R.1575. pQadd2 was constructed by annealing and PCR extending oligonucleotides 2ML9_1/2 encoding a double *lox* cassette (*lox511*/*66* plus *loxFAS*/*71*), and cloning the resultant product between the unique PstI and EcoRI sites of pAT19. The P3368-*FbFP* cassette was inserted into pQadd2 by PCR amplifying the *cphy3368* promoter region from *C. phytofermentans* genomic DNA using primers P3368_1/2 and cloning the product into the SpeI and XhoI sites between the *lox511*/*66* and *loxFAS*/*71* sites of pQadd2. The *PpFbFPm* gene was PCR amplified from pMTC6 (32) using primers P3368_FbFP_1/2 and cloned between the XbaI and XhoI sites directly downstream of P3368 to make pQadd2.P3368-FbFP. To enable cotransformation of pQadd2.P3368-FbFP and a *cre* plasmid into the same cell, pQcre2 was built by PCR amplifying the P*pagA*-*cre* cassette from pQcre1 using primers Cre2_1/2 and subcloning it into the unique EcoRI site of pMTL82351 (CHAIN Biotech). The sequences of all plasmids were confirmed by Sanger sequencing.

### Genomic insertion of *lox* sites.

The *C. phytofermentans* strains carrying *lox*7*1* and *lox*66 sites for prophage deletion were made by sequential conjugal delivery and curing of plasmids pQlox71R.2944 and pQlox66F.2993 (strain DI-AS) or plasmids pQlox71F.2944 and pQlox66F.2993 (strain DI-SS). Following delivery of each plasmid, we confirmed the intended targetron insertion in 10 transconjugants by PCR using primers flanking the insertion site, cured the plasmid from 3 transconjugants, and demonstrated plasmid loss by PCR and restoration of erythromycin sensitivity. Following delivery and curing of plasmids for both *lox* sites, PCR and sequencing confirmed the presence and orientation of both the *lox71* site at 591 bp from the *cphy2944* start in strain DI-AS and at 526 bp from the *cphy2944* start in strain DI-SS (primers OK_2944_1/2). Similarly, the location and orientation of the *lox66* site at 177 bp from the *cphy2993* start were confirmed in both strains (OK_2993_1/2). The *C. phytofermentans* int1575 strain was made by conjugal delivery and curing of pQadd1R.1575. The presence and antisense orientation of the *lox511*/*71*_*loxFAS*/*66* cassette at 96 bp from the start of *cphy1575* in the int1575 genome was confirmed in 8 of 10 transconjugants by PCR and sequencing (OK_1575_1/2).

The absence of additional, off-site genomic targetron insertions in strains DI-SS, DI-AS, and int1575 was confirmed by inverse PCR (27). To this end, genomic DNA was extracted and purified from 5 ml of log-phase culture using the GenElute Bacterial Genomic DNA kit (NA2110; Sigma). DI-SS and DI-AS genomic DNA (1 μg) was digested with HindIII; int1575 genomic DNA was digested with EcoRI. Digested DNA was purified with QIAquick PCR purification kit (28104; Qiagen) and ligated with T4 DNA ligase (M0202S; NEB). Intron insertion sites were PCR amplified by Q5 polymerase (M0491; NEB) using primers int_1/2 with a 7-min extension time. Targetron insertion sites were identified by gel extraction and sequencing of the PCR products.

### Genomic deletion and insertion using Cre recombinase.

To effectuate Cre-mediated genomic deletions, pQcre1 was conjugated into *C. phytofermentans* strains DI-SS and DI-AS, and its presence in 10 transconjugants was confirmed by PCR (primers pAMB1_1/2). Liquid cultures of the ten transconjugants were grown in liquid medium containing erythromycin, and deletion of the 38.9-kb region between the *lox71* and *lox66* sites was diagnosed using 3 PCRs: two reactions to confirm the absence of products using primers 2944_1/2 and 2993_1/2 and a reaction to amplify a product spanning the deleted region using primers 2944_1/2993_2. pQcre1 was cured from Del-SS and Del-AS as described above.

To make a Cre-mediated genomic insertion by RMCE, pQadd2.P3368-FbFP and pQcre2 were sequentially conjugated into strain int1575, and the simultaneous presence of both plasmids in 10 transconjugant colonies was confirmed by PCR using primers pBP1_1/2 for pQcre2 and pAMB1_1/2 for pQadd2.P3368-FbFP. After 1 transfer in medium containing erythromycin and spectinomycin, PCR using primers OK_1575_1/2 diagnosed insertion of the P3368*-FbFP* cassette into the int1575 genome, yielding strain int1575_FbFP.

### Measurement of FbFP fluorescence.

Cellular fluorescence was compared between triplicate cultures of *C. phytofermentans* WT and strain int1575_FbFP. Cultures were grown to log phase (OD_600_ of 0.6) in glucose or cellobiose medium. One milliliter of culture was centrifuged, and the cell pellet was washed once in phosphate-buffered saline PBS and resuspended in 500 μl PBS. The OD_600_ of each culture was confirmed, and the cellular fluorescence was measured in black 96-well Costar plates using a Safas Xenius XMA (excitation, 448/20 nm; emission, 495/20 nm; photomultiplier, 850 V). Background fluorescence of PBS was subtracted from measurements, and the fluorescence measurements were normalized to culture density (OD_600_).

### Data availability.

Plasmids from this study have been submitted to the Addgene Plasmid Repository and are available using the accession numbers listed in Table 1.
